# The importance of urgency in decision making based on dynamic information

**DOI:** 10.1371/journal.pcbi.1009455

**Published:** 2021-10-04

**Authors:** Lorenzo Ferrucci, Aldo Genovesio, Encarni Marcos

**Affiliations:** 1 Department of Physiology and Pharmacology, Sapienza University of Rome, Rome, Italy; 2 Instituto de Neurociencias de Alicante, Consejo Superior de Investigaciones Científicas–Universidad Miguel Hernández de Elche, Sant Joan d’Alacant, Spain; University of Groningen, NETHERLANDS

## Abstract

A standard view in the literature is that decisions are the result of a process that accumulates evidence in favor of each alternative until such accumulation reaches a threshold and a decision is made. However, this view has been recently questioned by an alternative proposal that suggests that, instead of accumulated, evidence is combined with an urgency signal. Both theories have been mathematically formalized and supported by a variety of decision-making tasks with constant information. However, recently, tasks with changing information have shown to be more effective to study the dynamics of decision making. Recent research using one of such tasks, the tokens task, has shown that decisions are better described by an urgency mechanism than by an accumulation one. However, the results of that study could depend on a task where all fundamental information was noiseless and always present, favoring a mechanism of non-integration, such as the urgency one. Here, we wanted to address whether the same conclusions were also supported by an experimental paradigm in which sensory evidence was removed shortly after it was provided, making working memory necessary to properly perform the task. Here, we show that, under such condition, participants’ behavior could be explained by an urgency-gating mechanism that low-pass filters the mnemonic information and combines it with an urgency signal that grows with time but not by an accumulation process that integrates the same mnemonic information. Thus, our study supports the idea that, under certain situations with dynamic sensory information, decisions are better explained by an urgency-gating mechanism than by an accumulation one.

## Author summary

Decisions are the result of a deliberative process that assesses the suitability of each potential option. However, the specific dynamics of such process are still under debate, with two influential views. On the one hand, a standard view on perceptual decision making proposes that sensory evidence in favor of each option is sequentially sampled and accumulated until the accumulation reaches a bound and a decision is made. On the other hand, an alternative view proposes that, once sampled, instead of accumulated, sensory evidence is low-passed filtered and weighted by an urgency signal, which increases with time. Here using a perceptual decision-making task with dynamic sensory evidence and two computational models, related with each view, we wanted to distinguish whether decisions can be better described by the standard proposal or by the alternative one. Our results show that a model with a low-pass filter and an urgency signal can describe the experimental data better than a model of accumulation, even in a situation where sensory evidence sequentially disappears. Our study contributes to describe the decision-making process by providing experimental and computational support for one of the most influential views in decision making.

## Introduction

When making decisions, one needs to predict which option will lead to the best outcome. To do that, information is gathered from all possible sources and weighted according to its reliability. In laboratory studies, this has been investigated using perceptual decision-making tasks requiring sensory evidence discrimination to correctly select between two options [1,2]. Neurons from the frontoparietal network exhibit a ramping activity that seems to mimic the deliberative process of decision making [2–6]. The general agreement is that, during such deliberative process, information is sequentially sampled until a bound is reached and a decision is made. However, how such samples are incorporated into the decision-making process is still open to debate. Here, we address this issue by studying the accuracy of two widely accepted alternative models–the Evidence Accumulation Model and the Urgency Gating Model—to describe experimental data collected from a decision-making task with information that varied over time.

In the last decades, two alternative theories have been proposed to explain decision making. The standard view proposes that decisions are the result of accumulating evidence until a threshold is reached. This view has led to the development of the Evidence Accumulation Model (EAM), which has accounted for a variety of behavioral and neuronal data [7–13]. However, recently, this view has been questioned by an alternative theory that proposes that, rather than being accumulated, sensory evidence is weighted by an urgency signal that grows with time. This could also explain the ramping activity of neurons in the decision-making network as well as behavior in different decision-making paradigms [14–17]. Within this view, the Urgency Gating Model (UGM) proposes that evidence is low-pass filtered and multiplied by a temporally increasing signal [15]. Thus, previous research supports both kinds of models. However, in most tasks, decisions relayed on constant information and, although they have proven to be valid to discriminate between the models in some cases [18], in some others, they have proven to be inadequate [15,19].

In the last years, new perceptual decision-making paradigms involving changes of information in the course of a trial have been proposed [15,16,20–24]. In such tasks, perceptual evidence is sequentially presented in favor of one of two options and humans or animals have to decide which one of the two options is the most favored one. Although these tasks have provided a significant advance towards the description of a general mechanism for perceptual decision making, the question of whether the neuronal dynamics during such decisions follow the mechanism proposed by either the EAM or the UGM remains still unanswered. To distinguish between the models, Cisek et al. [15] used a perceptual decision-making task, called the tokens task, in which visual stimulus sequentially jumped towards a right or left target and stayed there until subjects committed to a choice. In these cases, the EAM failed to explain the experimental data and, instead, the UGM provided a reliable mechanism by which decisions might be made. The UGM model proposes that, rather than integrated, sensory evidence is modulated by an urgency signal that increases over time, reflecting the increasing need to make a decision as time passes [15,16]. Subsequently, by using the same task, Thura et al. [5] showed that the activity of the neurons in the premotor and primary motor cortex reflected the combination of sensory evidence with an urgency signal. However, the fact that novel sensory evidence remained available until the decision was made might have biased their results, since there was no implicit need for integration. In other words, one could assess the situation just before the decision is made and obtain the same information that one would get by observing for the entire period. Thus, there is still no real consensus on whether the ramping activity of the neurons in the frontoparietal network reflects the integration of sensory evidence or is instead the result of a combination of sensory evidence with an urgency signal that increases over time.

**Fig 1:**
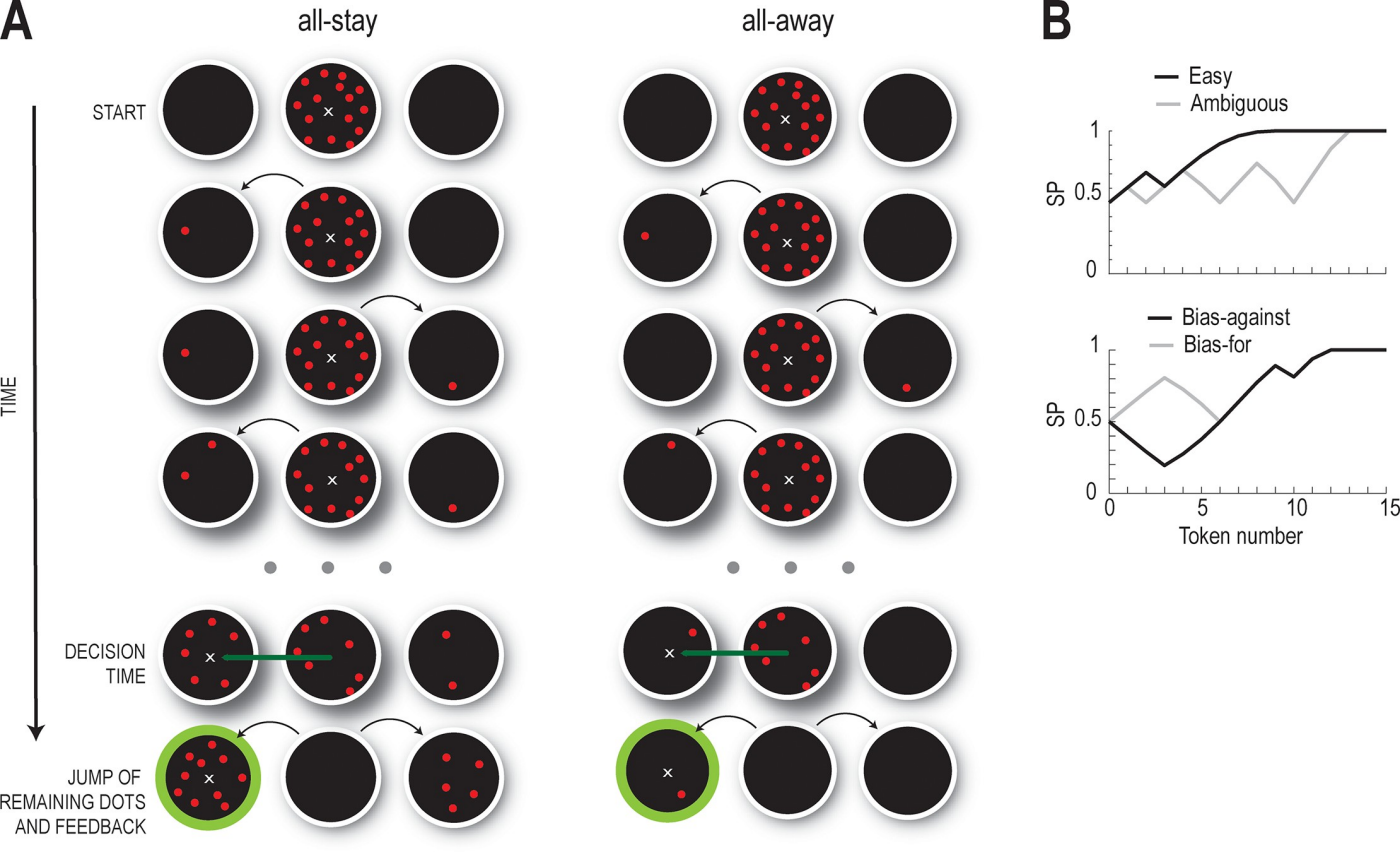
Experimental design. **(A)** Temporal presentation of events during a trial. Each trial starts with all the tokens in the central circle. After the participant moves the cursor to inside the central circle, the tokens start jumping successively to the other two (target) circles. In the all-stay trials the tokens remain visible after jumping, while in the all-away trials they disappear soon after they have jumped. The participant has to guess which of the two target circles will contain more tokens at the end of the trial. **(B)** Success probability profiles for specific trial types. *Top panel*, success probability for easy (black) and ambiguous (gray) trials. *Bottom panel*, success probability for bias-against (black) and bias-for (gray) trials.

Here, we further contrast the predictions produced by the two computational models by using a modified version of the tokens task [5,15], introducing an additional condition in which novel sensory evidence is removed from the screen soon after it is provided. In each trial, fifteen tokens, presented in a central circle, sequentially jumped towards a left or right target, indicated by a circle on the screen. Subjects had to guess which target would have more tokens by the end of the trial. They could make their choice at any time. The trials were divided into blocks that contained only “all-stay” or “all-away” trials (Fig 1A). In all-stay trials the tokens stayed visible during the entire trial whereas in the all-away condition they disappeared soon after they jumped into one target (see Materials and Methods). With this new task design, we can test both models under conditions that might favor evidence integration.

## Results

Overall, the subjects performed the task with an accuracy greater than chance in both the all-stay and the all-away conditions, although with a significantly higher accuracy in the all-stay trials (73 ± 2%) than in the all-away trials (68 ± 2%; paired-samples *t*-test, p = 0.04, *t* = 2.25). In addition, their decision times (DTs) were slower in the all-stay than in the all-away condition, at 1.465 ± 0.074 s and 1.207 ± 0.121 s, respectively (paired-samples *t*-test, p = 0.04, *t* = 2.25). The subjects’ mean (± standard error of the mean [SEM]) baseline reaction time (RT) used to calculate the DTs was 0.347 ± 0.009 s.

### Behavior is modulated by context

**Fig 2:**
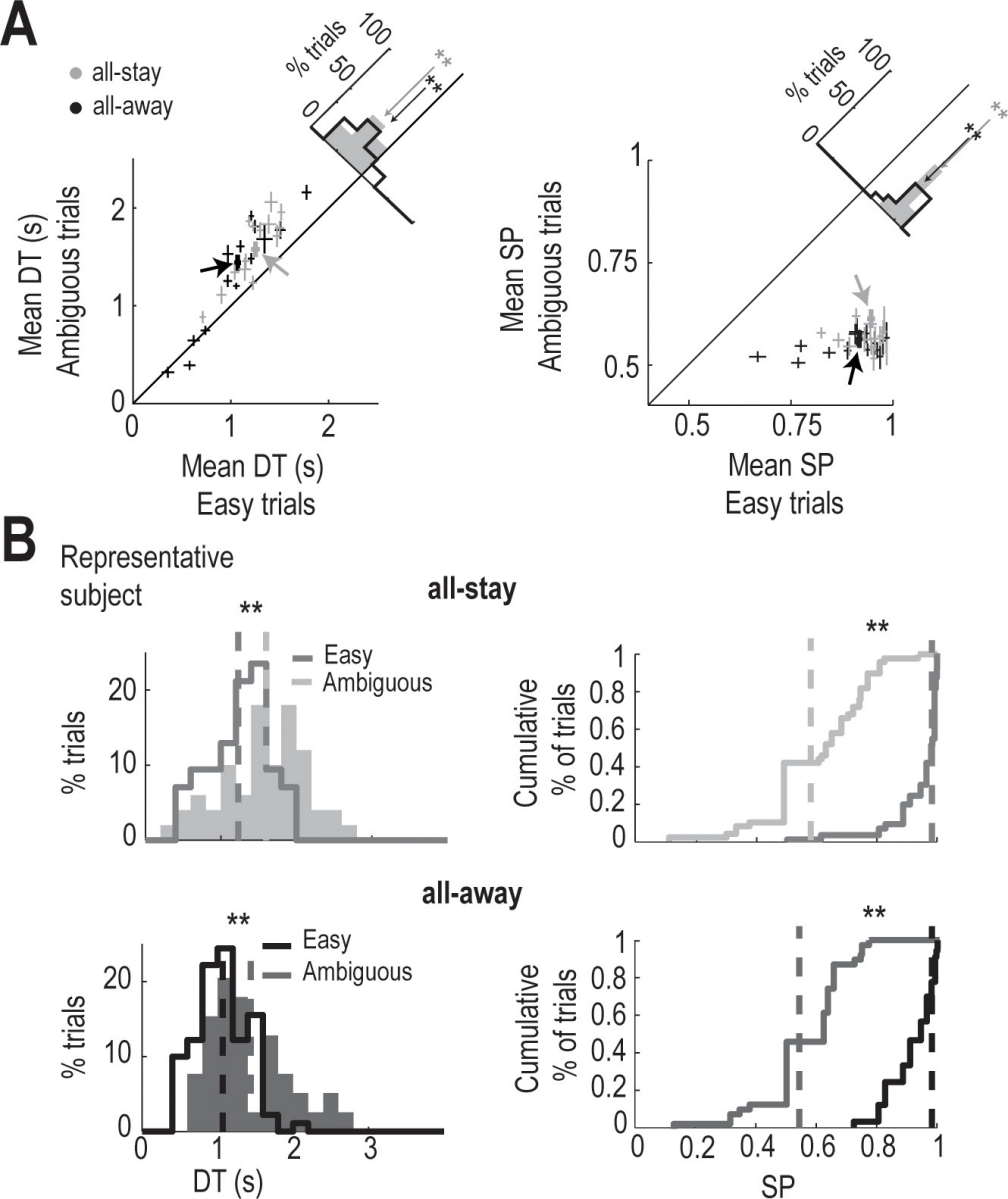
Behavior of subjects during easy and ambiguous trials. **(A)** *Left panel*, individual mean decision times (DTs) observed during easy and ambiguous trials for all-stay (gray) and all-away (black) conditions. *Inset panel* shows a histogram with the difference in DTs between trial types within each condition. The DTs for easy and ambiguous trials were significantly different in both conditions (n = 15; paired-samples *t*-test, ** p < 0.001; all-stay, p = 0.000003, *t* = 7.48; all-away, p = 0.0007, *t* = 4.32). *Right panel*, success probability (SP) at decision time for easy and ambiguous trials. *Inset panel* shows a histogram with the difference in SPs for the two trial types within each condition. The difference is significant for both conditions (n = 15; paired-samples *t*-test, ** p < 10^−8^; all-stay, p = 0.4 × 10^−12^, *t* = 25.58; all-away, p = 0.2 × 10^−9^, *t* = 16.38). Error bars indicate SEM. **(B)** DTs (*left panels*) and SPs (*right panels*) of a representative subject, whose mean DT and SP values are indicated by arrows in (A). The subject clearly shows the same behavioral effect as was observed for the group: faster DT and higher SP at decision time for easy trials than for ambiguous trials in both all-stay and all-away conditions (Kolmogorov–Smirnov test, ** p < 0.01; n_easy_ = 52, n_ambiguous_ = 37, all-stay: DTs, p = 0.00005, *D* = 0.4; SPs, p = 0, *D* = 0.89; n_easy_ = 28, n_ambiguous_ = 22, all-away: DTs, p = 0.002, *D* = 0.35; p = 0, *D* = 0.97).

We used the easy and ambiguous trials to investigate whether the subjects’ performance was influenced by the type of trial. DTs and success probabilities (SPs) in these two trial types indicated that the subjects behaved differently in easy and ambiguous trials in both the all-stay and all-away conditions (Fig 2A). Specifically, DTs were faster and SPs higher for easy than for ambiguous trials in both conditions (mean DT and SP ± SEM for all-stay: 1.587 ± 0.088 s and 57 ± 1% for ambiguous trials, 1.228 ± 0.058 s and 93 ± 1% for easy trials; mean DT and SP ± SEM for all-away: 1.331 ± 0.146 s and 55 ± 1% for ambiguous trials, 1.049 ± 0.095 s and 89 ± 2% for easy trials). Fig 2B shows the behavior of one representative subject during ambiguous and easy trials in all-stay and all-away conditions. Consistent with the group’s behavior, this subject responded significantly faster in easy trials than in ambiguous trials in both experimental conditions (all-stay: 1.578 ± 0.080 s for ambiguous trials and 1.251 ± 0.044 s for easy trials; all-away: 1.440 ± 0.082 s for ambiguous trials and 1.069 ± 0.034 s for easy trials) and his/her SPs were significantly higher for easy trials than for ambiguous trials (all-stay: 61 ± 2% for ambiguous trials and 95 ± 1% for easy trials; all-away: 56 ± 2% for ambiguous trials and 92 ± 1% for easy trials).

Next, we investigated the subjects’ performance during bias-against and bias-for trials. These two trial profiles are the most interesting of the study because, for non-leaky sensory evidence (all-stay condition), the decision-making models make different predictions about DTs for these trial types [15]. While the UGM predicts no differences between them, the EAM predicts that DTs in bias-against trials will be longer than those in bias-for trials. Importantly, using only the all-stay condition, Cisek et al. [15] showed that the subjects’ DTs did not differ between bias-against and bias-for trials, providing strong evidence in support of the UGM. However, one possible explanation for their results was that accumulation or integration of evidence was not required by the task because the information was available on the screen during the entire trial, favoring urgency dynamics. Our all-away condition was designed to control for that possibility. By using a condition in which each token disappeared from the screen after jumping, we could control for the possibility that the previous findings in favor of the UGM over the EAM were not merely a consequence of the limitations of the original experimental design.

**Fig 3:**
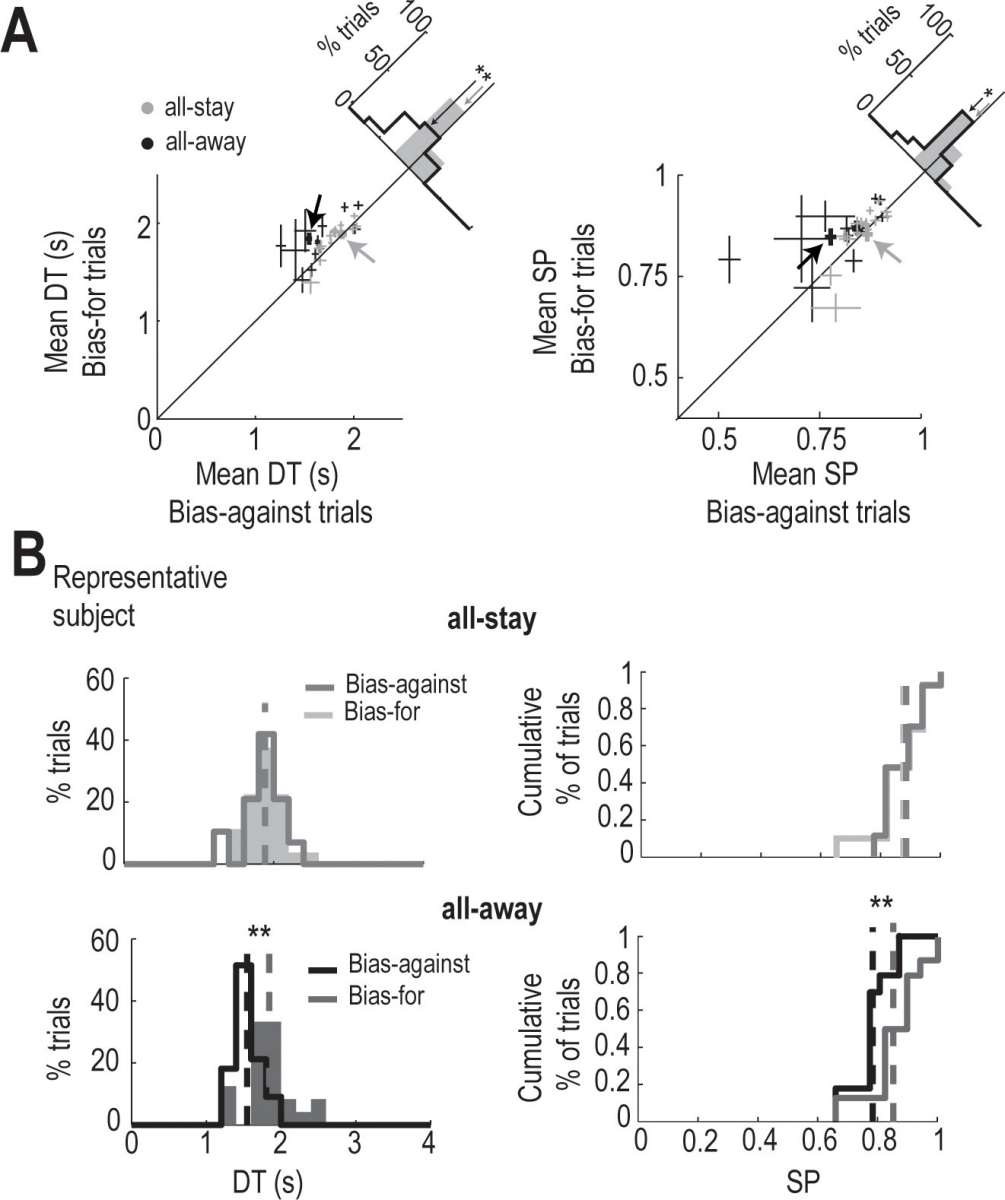
Behavior of subjects during bias-against and bias-for trials. (A) *Left panel*, DTs observed during bias-against and bias-for trials in all-stay (gray) and all-away (black) conditions. *Inset panel* shows a histogram with the difference in DTs between the two trial types within each condition. The difference in DTs between bias-against and bias-for trials is significant in the all-away condition but not in the all-stay condition (n = 15; paired-samples *t*-test, ** p < 0.01; p = 0.001, *t* = 3.96). A Bayes Factor of 16.929 (> 3) in the all-away condition and of 0.249 (< 1/3) in the all-stay condition confirm the results. *Right panel*, SPs at decision time for bias-against and bias-for trials. *Inset panel* shows a histogram with the difference between SPs for the two trial types within each condition. The difference is significant only in the all-away condition (n = 15; paired-samples *t*-test, * p < 0.05; p = 0.01, *t* = 2.78). A Bayes Factor of 4.063 (> 3) in the all-away condition and of 0.277 (< 1/3) in the all-stay condition support the result. Error bars indicate SEM. **(B)** DTs and SPs for the same subject as in Fig 2B, whose mean DT and SP values are indicated with arrows in (A). The subject exhibits the same behavioral effect as was observed for the group: the DT and SP only differed significantly between bias-against and bias-for trials in the all-away condition (n_bias-against_ = 33, n_bias-for_ = 24; Kolmogorov–Smirnov test, ** p < 0.001; DTs, p = 0.00002, *D* = 0.62; SPs, p = 0.0001, *D* = 0.57).

We, first, examined the results of the all-stay condition trials. Consistent with Cisek et al. [15][23], we observed no differences in the subjects’ mean DT or SP (± SEM) between bias-for and bias-against trials (bias-against: 1.810 ± 0.036 s and 85 ± 1%; bias-for: 1.845 ± 0.043 s and 86 ± 2%; Fig 3A). Then, we examined the results of the new condition that we had introduced: the all-away condition. Interestingly, the subjects’ mean DT and SP differed significantly between the two trial types (bias-against: 1.650 ± 0.056 s and 80 ± 2%; bias-for: 1.826 ± 0.053 s and 86 ± 1%; Fig 3A). Notably, contrary to the prediction of the EAM for non-leaky sensory evidence (see text above), the mean DT was shorter in bias-against trials than in bias-for trials. Indeed, the behavioral difference between both types of trials is related to shortened DTs observed in the bias-against trials compared with the same trials in the all-stay condition. The shortened DTs reduces accuracy but not significantly (paired-samples t-test p = 0.25; all-away: 60 ± 6%; all-stay: 68 ± 6%). Fig 3B shows the DTs and SPs of the same subject represented in Fig 2B. This subject showed no difference in DTs and SPs between bias-against and bias-for trials in the all-stay condition (bias-against: 1.883 ± 0.048 s and 87 ± 1%; bias-for: 1.8883 ± 0.050 s and 85 ± 2%), but did show a significant difference between trial types in the all-away condition (bias-against: 1.546 ± 0.030 s and 78 ± 1%; bias-for: 1.841 ± 0.063 s and 85 ± 2%), with longer DTs and higher SPs in bias-for than for bias-against trials.

Consistent with previous research [15,16], subjects were biased towards an urgency-like strategy in the all-stay condition. Next, we wondered whether subjects’ behavior in the all-away condition might be influenced by the order in which they performed the two conditions (see Materials and Methods). In other words, did subjects’ behavior in the all-away condition depend on whether they encountered first the all-stay condition than the all-away condition or vice versa? To answer this question, we analyzed DTs and SPs of subjects sorted by whether they belonged to the first or the second group. Mean DT and SP (± SEM) in bias-for and bias-against trials were close to significance when subjects performed the all-away condition after the all-stay condition (n = 8, paired-samples t-test p = 0.051 for DTs and p = 0.164 for SPs; bias-for: 1.73 ± 0.06 and 83 ± 2%; bias-against: 1.56 ± 0.05 s and 78 ± 4%) and significantly different when they performed the all-away condition at first (n = 7, paired-samples t-test p < 0.05; p = 0.0152, t = 3.36 for DTs and p = 0.0171, t = 3.26 for SPs; bias-for: 1.94 ± 0.07 s and 89 ± 2%; bias-against: 1.75 ± 0.09 s and 83 ± 3%). Nevertheless, in both cases, mean DT and mean SP were longer and higher, respectively, in the bias-for than in the bias-against trials, indicating no influence of the order of blocks in behavior.

### The urgency-gating model correctly predicts behavior

**Fig 4:**
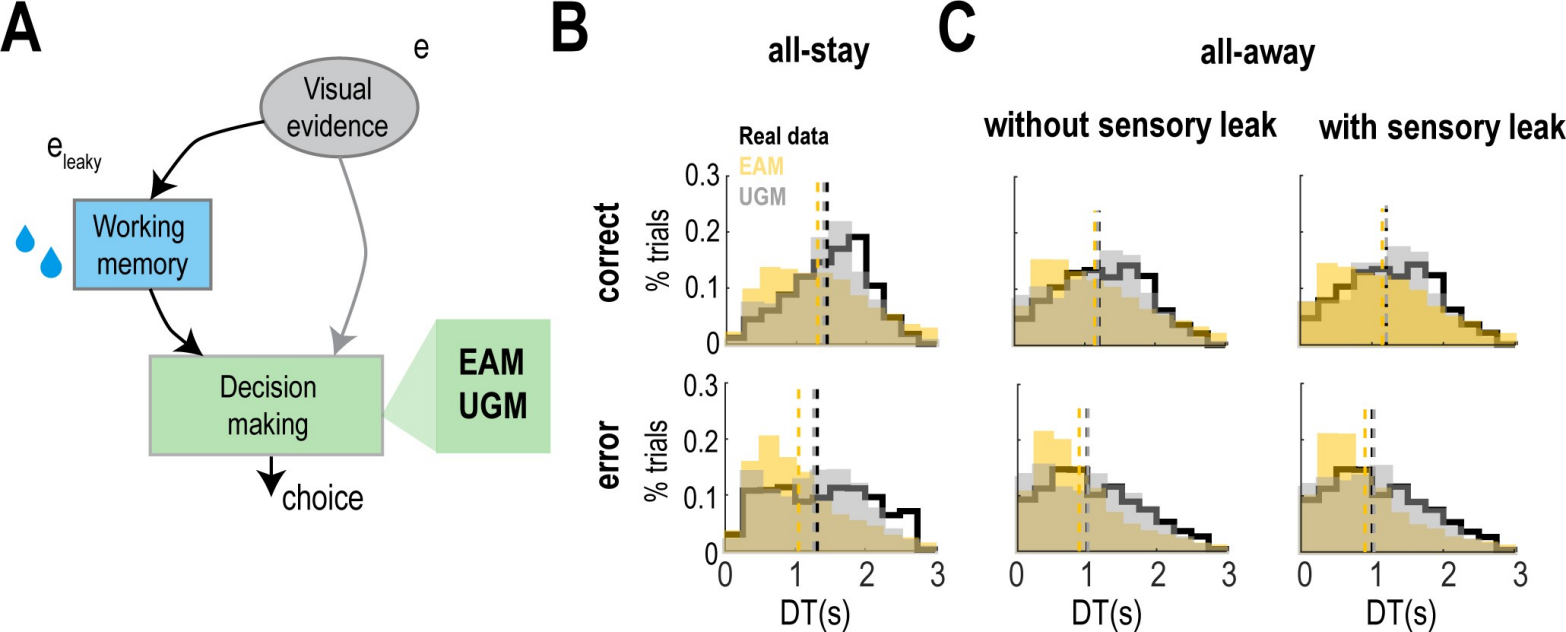
Computational framework and model simulations for correct and error trials. **(A)** Schematic diagram of the complete network that simulates the observed experimental results. Visual evidence is either provided directly to the decision-making module or stored in a working memory that then provides the information (*e*_leaky_) to the decision-making module. The information (*e*_leaky_ or *e*) is used by the EAM or the UGM to make a choice. **(B)** Distributions of DTs in correct and error trials in the all-stay condition when all individual trials are pooled together for real data and simulated EAM and UGM. **(C)** Same conventions as in (B) for the all-away condition. In this case, simulations were performed with and without leakage in the sensory evidence.

We investigated whether the behavioral results could be better explained by the EAM or by the UGM. To do that, we devised a computational framework in which sensory evidence directly fed the decision-making model, implemented as the EAM or the UGM, or did so through a working memory module (Fig 4A), simulating the all-stay and all-away conditions, respectively. Therefore, the working memory was responsible for monitoring and remembering the sensory evidence that disappeared from the screen. To fit the data with the models, we used a differential evolution algorithm with the experimental DTs recorded in correct and error easy, ambiguous, bias-for, and bias-against trials (see Materials and Methods). Table 1 shows the mean and SEM of the best fitting parameters that were obtained for each model and condition. In the all-stay condition, DTs in correct and error trials were better fitted by the UGM than by the EAM (Fig 4B), as indicated by a lower difference between mean experimental and simulated DTs for both kinds of trials (EAM: 135 ms for correct trials, 279 ms for error trials; UGM: 51 ms for correct trials, 41 ms for error trials). Moreover, better performance of the UGM over the EAM was observed when the experimental data was fitted in the all-away condition (Fig 4C), with lower difference in mean DTs that held for sensory evidence without and with leakage (EAM without sensory leak: 79 ms for correct trials, 134 ms for error trials; EAM with sensory leak: 72 ms for correct trials, 115 ms for error trials; UGM without sensory leak: 14 ms for correct trials, 12 ms for error trials; UGM with sensory leak: <1 ms for correct trials, 26 ms for error trials). Indeed, in the two experimental conditions, the shapes of the distributions were better estimated by the UGM in all cases, with the EAM tending to predict shorter DTs resulting in positive skew distributions.

**Table 1:** Mean and SEM across subjects of the best fitting parameters for the EAM and the UGM in the all-stay and all-away conditions with and without leakage in the sensory evidence.

			ν	η	θ	L_e_
**all-stay**	**DDM**	**Mean**	0,04456	0,00478	0,29062	
		**SEM**	0,00183	0,00140	0,01289	
	**UGM**	**Mean**	3,76432	7,65377	23022,92488	
		**SEM**	0,61932	3,35522	1124,45466	
**all-away without sensory leak**	**DDM**	**Mean**	0,03915	0,01345	0,25270	
		**SEM**	0,01519	0,01464	0,06876	
	**UGM**	**Mean**	4,12036	12,84759	18791,13380	
		**SEM**	2,55487	20,17686	6304,48655	
**all-away with sensory leak**	**DDM**	**Mean**	0,06918	0,01751	0,26127	0,12341
		**SEM**	0,00654	0,00319	0,01986	0,04727
	**UGM**	**Mean**	5,05699	13,03403	17660,64882	0,21371
		**SEM**	0,64309	5,08468	1736,69700	0,04929

**Fig 5:**
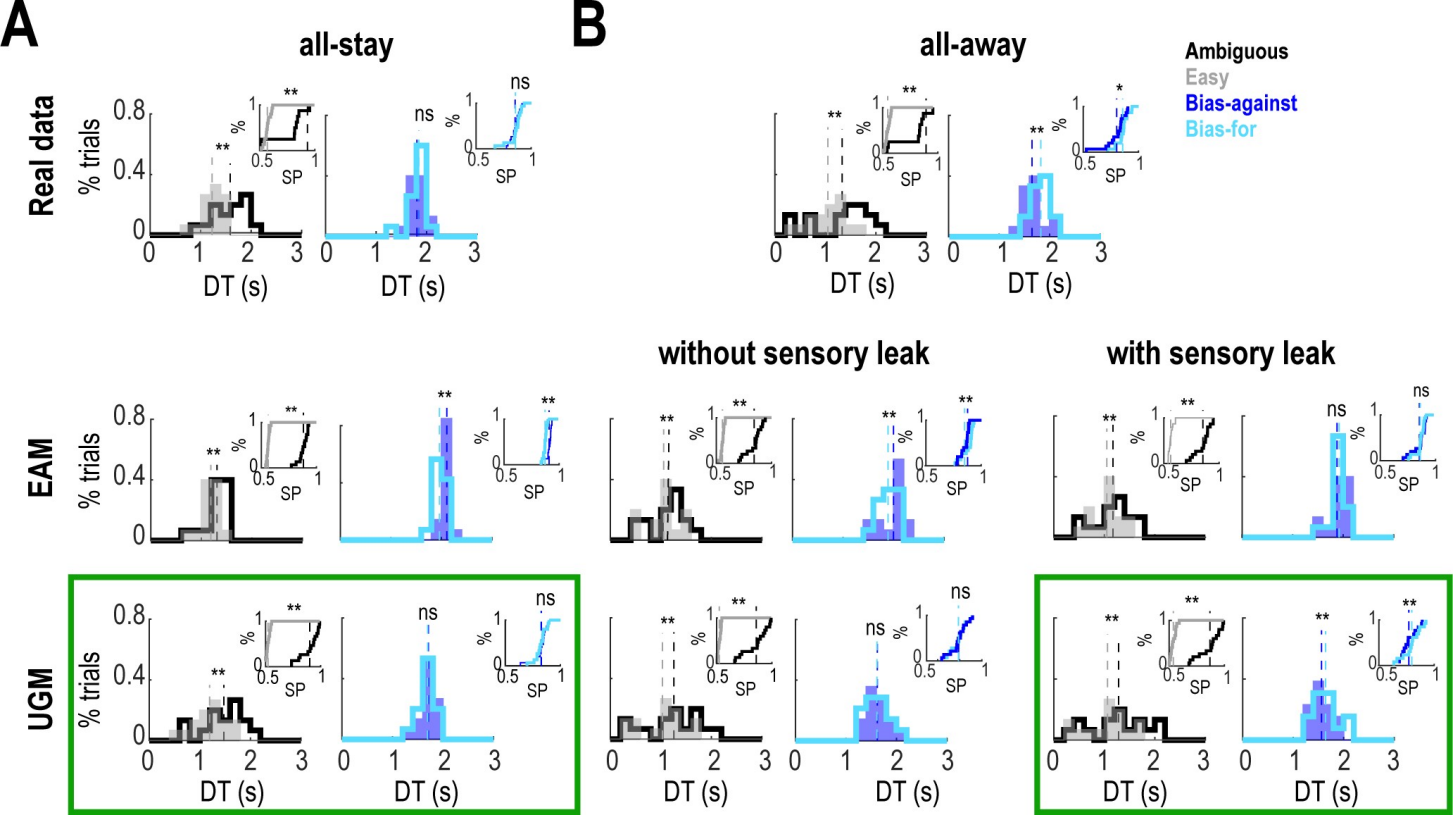
Model simulations for different trial types. **(A)** Distributions of DTs for ambiguous, easy, bias-for, and bias-against trials in real and simulated data in the all-stay condition. *Inset panel*, Cumulative distributions of SPs for each trial type. Differences in DTs and SPs in ambiguous and easy trials are fitted correctly by the EAM and the UGM (paired-samples *t*-test, ** p < 0.01). Lack of difference in DTs and SPs in bias-for and bias-against trials is only fitted by the UGM (paired-samples *t*-test, ** p < 0.01, ns: not significant). Dashed lines indicate the mean of the distributions. Green rectangle shows the results that are consistent with the real data. **(B)** Same conventions as in (A) for the all-away condition with simulations performed without and with leakage in the sensory evidence. Differences between DTs and SPs in easy and ambiguous trials are fitted correctly by the EAM and the UGM without and with sensory leakage (paired-samples *t*-test, ** p < 0.01). Longer DTs and higher SPs in bias-for than in bias-against trials are only correctly fitted by the UGM when sensory evidence leaks away (paired-samples *t*-test, * p < 0.05, ** p < 0.01, ns: not significant).

We then investigated the data obtained with the models for each trial type separately to assess whether the models showed the same effect as observed in the experimental data. As previously shown [15], in the all-stay condition, the EAM correctly produced shorter DTs and higher SPs in easy than ambiguous trials but predicted longer DTs and higher SPs in bias-against trials compared with bias-for trials (Fig 5A), despite no difference was observed in the experimental data in those kinds of trials. On the contrary, the UGM correctly replicated the same effects in DTs and SPs for the four kinds of trials, with distributions that were very similar to those from the real data (Fig 5A, green square). Next, we looked at the all-away condition in two cases: when the sensory evidence had no leak (L_e_ = 0) and when it leaked away with time (L_e_>0) (see Materials and Methods). In the simulations without sensory leak, both models failed to correctly reproduce the experimental data and showed the same results as those obtained for the all-stay condition. In other words, both models correctly reproduced the differences in DTs and SPs in easy and ambiguous trials but failed to produce shorter DTs and lower SPs in bias-against than bias-for trials, with an opposite result in the EAM and no difference in the UGM (Fig 5B). However, a leak in the sensory evidence (L_e_>0) was sufficient for the UGM to explain the experimental data in the four types of trials, but not for the EAM, which predicted no significant difference in DTs between bias-for and bias-against trials (Fig 5B). In addition, the shapes of the distributions of DTs and SPs in the real and UGM data were comparable. Thus, the UGM is capable of explaining the data in the two experimental conditions of our task.

The fitted parameters of the UGM seem to indicate a difference in the strategy used in the two experimental conditions: higher mean drift rate and lower boundaries were estimated in the all-away condition compared to the all-stay condition (Table 1). This result could be related to an increase in the need to make a decision when sensory evidence disappears, related to the gradual “forgetting” of information. Nevertheless, both experimental conditions support the existence of an urgency signal that modulates the decision-making process, strengthening the idea of decisions being the result of sensory evidence (visual or mnemonic) combined with an internal urge to decide.

## Discussion

In this work we have advanced in the understanding of the general mechanism of decision making. To accomplish this, we used an experimental task in which the available sensory information varied over time. Changing information over time appears to be a critical element of task design, since it allows a more efficient discrimination between computational models of decision making than that using tasks with constant information [19], and therefore between decision-making mechanisms. In our experimental task, fifteen tokens, presented in the center of the screen, successively jumped into a circular target either to their right or to their left. Subjects were required to decide which of the two targets would contain more tokens at the end of the trial. They could make their choice at any time, but to discourage random guessing, they were required to reach a specific number of correct trials to finish the experiment. This task represents a modification of a previously described tokens task [15,16], in which we have introduced a new condition that required the use of working memory and that could favor the integration of sensory evidence. This addition has allowed us to compare decision making under conditions where each novel piece of sensory information was visually available until the end of the decision-making process (and thus did not explicitly require the integration of information) with conditions where novel sensory evidence was removed shortly after it had been presented (thus requiring the active maintenance of information in the brain). With this comparison, we have advanced in the description of the general mechanism by which decisions are made under different circumstances. We have shown that the experimental results observed in both contexts could be explained by a decision-making model that low-pass filters sensory evidence, provided by a visual-input or a working-memory module, and multiplies it by an urgency signal. In this model, the decision is made when the result of the multiplication reaches a decision threshold.

We have focused our study on the two main models in the literature of the mechanism governing decision making: the EAM and the UGM. In our behavioral trials, sensory evidence was represented by the number of tokens that had already jumped to each target. This approach is different from the one previously used [15], in which the success probability for one target was calculated after each tokens’ jump and used as sensory evidence. The reasons we used this alternative approach are twofold. First, estimating the number of tokens in each target is easier for subjects than estimating the probability of success for each target. Indeed, our approach allows for a simpler and more realistic way of representing and updating sensory evidence, especially when it needs to be maintained in memory, which does not rely on relatively complex calculations of probabilities. Second, the predictions of each model are equivalent to when using success probability.

One possible explanation for the fact that our results support the UGM over the EAM might be that the EAM is fundamental for non-noisy sensory evidence, such as the stimuli provided in a motion-direction discrimination task, but not for non-noisy sensory evidence [14,16,17,25–30]. However, using a paradigm similar to the tokens task but with noisy stimuli, Thura et al. [16] showed that, even in such cases, when the available information changes over time, the EAM cannot account for the behavioral observations while the UGM can. Thus, the decision-making mechanism for noisy or non-noisy sensory evidence appears to be the same.

The EAM has been the standard view in the decision-making literature for many years but recently it has been questioned by models of urgency and of accumulation with time-varying boundaries [15,16,31–33]. Here we implemented the UGM, which relies on an urgency signal that grows with time and that can resolve situations of high time-pressure. An alternative to that is offered by the time-varying boundaries models that propose that decision boundaries decay with time. In both cases, less sensory or accumulated evidence, respectively, is needed to make a decision as time passes. We have shown that the UGM can explain our experimental data better than the EAM. The same result would be expected when comparing the UGM with a time-varying boundaries model. The reason is that these models only differ from the EAM in the decision boundaries, which are fixed in the latter case and time-varying in the former. Thus, evidence accumulation in bias-for and bias-against trials will reach the decision boundary at similar time points, independently of whether the boundary keeps fixed or decays with time. Further additions to the model would be necessary to account for our data, significantly increasing the complexity of the model.

Using a random motion discrimination task [25–29], Winkel et al. [24] showed that early evidence influenced subsequent decisions. This was considered as an evidence in favor of the EAM and against the UGM. However, the authors missed a low-pass filter in the implementation of the UGM and, indeed, when such filter, with a short time constant (250 ms), was added to the model, the UGM could correctly fit the data [17,34]. Using a similar task, Evans et al. [23] showed that participants were faster and more accurate in their responses when early pulses of motion were consistent with the subsequent direction of motion than when they were inconsistent. Moreover, the behavioral data was better explained by the EAM than by the UGM. Instead, in our all-away condition, we found an opposite result, i.e. decision times were longer when an early pulse was consistent with the subsequent evidence (bias-for) than when it was not (bias-against) and the results could be better explained by the UGM than by the EAM. In an early study, we showed that the effect of pulses in participants’ behavior depended on the decision policy employed, which could be explained by different slopes of the urgency signal [34]. This is, indeed, consistent with the lack of effect observed in Evans et al. [23] for responses longer than 2 s and a possible explanation for the behavioral differences between their study and ours. Moreover, a possible reason for the low performance of the UGM in fitting the data from Evans et al. [23] might be the use of a non-optimal value of slope for the urgency signal, which was a fixed parameter during data fitting. Indeed, similar behavioral data was properly fitted by the UGM in previous studies [17,34].

Our results show that information in memory necessarily leaks away. Such leakage could be related to the arrival of new information or to the passage of time [35]. A recent study showed that the accuracy of subjects is unaffected by changes in the interval between two pulses of evidence, pointing to the arrival of new information as the main cause of memory leakage in a perceptual decision-making task [36]. Following this result, we assumed here that memory leakage occurs at the onset of each tokens’ jump and freezes after and before each jump. In future research, this could be formally investigated by varying the time between tokens’ jumps and by testing whether or not the difference in performance between bias-for and bias-against trials remains unaffected.

Perceptual decisions are influenced by factors that are irrelevant to the task and that can sometimes lead to a decrease in behavioral accuracy [37–40]. Yet two questions remain: (1) How are these factors integrated into the decision-making process?, and (2) Do such factors influence decisions made with constant and non-constant information in a similar way? Follow-up studies addressing these questions are needed to shed further light on the general mechanism of decision making by distinguishing between decision-making models.

Novel experimental tasks that can help to distinguish between decision-making models are essential for uncovering the underlying mechanisms of decision making. Moreover, the two main models for how decisions are formed—EAM and UGM—imply the involvement of different structures of the brain. Neurons in the lateral intraparietal cortex exhibit a ramping up activity that has been associated with the accumulation of sensory evidence when a decision is being made [2,14,41]. However, this interpretation has recently been questioned based on new experimental data showing that the ramping up activity might be an effect of averaging neural activity associated with instantaneous jumps at different times in different trials [42]. In other studies, neurons in the dorsal premotor cortex and primary motor cortex have been shown to combine sensory evidence with an urgency signal, with no sign of sequential evidence accumulation [5]. Furthermore, the basal ganglia have been identified as one part of the brain that controls the urgency of commitment [43]. Future studies should be designed to move the study of decision making from its general process to the investigation of the underlying specific contributions of each brain area.

## Materials and methods

### Ethics statement

All experimental procedures were in accordance with the ethical standards of the university research committee and with the Code of Ethics of the World Medical Association (Declaration of Helsinki, 1964) and its later amendments. The experimental protocol was approved by the Ethics Committee of the Physiology and Pharmacology Department at Sapienza University of Rome. All subjects provided written consent before participating in the experiment.

### Experimental task

Fifteen subjects performed the tokens task (aged 23–56, all right-handed, eight female). A 19.5” BenQ GL2023A LED monitor was used to display the visual stimuli for the task and a USB mouse was used as interface between the participants and the computer. The participants sat in front of the screen at a distance of approximately 60 cm. Our experimental protocol was based on the one proposed by Cisek et al. [15]. The main difference between the two was the addition of the “all-away” condition, described in detail below.

We used two trial conditions, which featured a similar sequence of events. At the beginning of each trial, three circles with white outlines, each 2.5 cm in diameter, appeared on the screen: one (central circle) was displayed at the center of the screen and the other two (targets) were placed 5 cm on either side of the central circle (Fig 1A). The central circle contained 15 dots (tokens) randomly distributed within its circumference. Subjects were required to place the cursor inside the central circle to start a trial. One of the tokens jumped from the central circle to one of the two targets every 200 ms. The subjects were required to select, by moving the mouse and placing the cursor inside one of the two peripheral circles, the target that they guessed would contain the majority of the tokens at the end of the trial. They could make their choice at any time before the last token jumped. Immediately after they had made their choice, the outline of the selected target changed from white to green, if the response was correct, or red, if it was incorrect. After the feedback, the remaining tokens jumped to one of the two targets every 20 ms, so a considerable amount of time could be saved by making decisions earlier. This motivated the subjects not to wait until the end of the trial to make their choice. An interval of 500 ms separated the end of one trial from the beginning of the next one.

We called the two trial conditions “all-stay” and “all-away”. In the all-stay condition, the tokens remained visible after jumping to one of the two targets [15]. Thus, the sensory evidence was always available to the subjects. Conversely, in the all-away condition, the tokens disappeared 200 ms after they jumped to one of the targets, so that sensory evidence was not available to the subjects for the remainder of the trial. The task was divided into twelve blocks, half of which comprised only all-stay trials and the other half only all-away trials. The subjects underwent all the blocks of one condition, followed by all the blocks of the other condition, and the order was alternated between subjects. To avoid random guessing, each block was complete only when the subject had achieved 70 correct answers.

The success probability (SP) of selecting target one over target two was calculated as [15]:

$$P(C|N_{1},N_{2},N_{r})=\frac{N_{r}!}{2^{N_{r}}}\sum_{k=0}^{min\;(N_{r,}7-N_{2})}\frac{1}{k!(N_{r}-k)!}$$

where *P*(*C*|*N*_1_,*N*_2_,*N*_r_) was the probability of being correct (*C*) when selecting target one when there were *N*_1_ tokens in target one, *N*_2_ tokens in target two, and *N*_r_ tokens still remaining in the central circle.

Within each of the 12 blocks, in both all-away and all-stay conditions, the direction in which the tokens jumped was randomly determined in 50% of the trials. In the other half of the trials, one of four predefined trial types was used: “easy” (15% of trials), “ambiguous” (15%), “bias-for” (10%), and “bias-against” (10%) [15]. In the easy trials, most of the tokens jumped only to the correct target, whereas in the ambiguous trials the tokens were evenly distributed between the two targets until just before the end (top panel of Fig 1B). The bias-for and bias-against trials were the most powerful for distinguishing between the decision-making models. The two cases differed only in the direction of jump of the first six tokens. In the bias-for trials, the first three tokens jumped to the correct target and the next three went to the incorrect target. The opposite occurred in the bias-against trials. In both cases most of the remaining tokens jumped to the correct target (bottom panel of Fig 1B). The random trials were included in order to prevent the subjects from predicting the pattern of the trials.

To estimate decision times (DTs), the subjects performed 40 additional trials in which only one token jumped to one of the two targets, which was randomly selected. The time from the arrival of the token in the target to the time at which the cursor of the mouse left the central circle and reached the chosen circle represented the baseline reaction time (RT) of the subject. The baseline RT of each subject was then subtracted from the RT obtained in the main task, which was computed as the difference between the time at which the cursor of the mouse left the central circle and the start of the trial. The SP was then computed at the estimated DT. Unless otherwise specified, all analyses were conducted using only correct trials.

### Computational models

#### Sensory evidence

The computational models used the same input (sensory evidence) to make a decision. The variation in the sensory evidence was estimated as:

$$\frac{de}{dt}=\delta_{t,t_{R}}-\delta_{t,t_{L}}$$ (1)

where the delta functions defined the tokens’ jump to either the right (δt_R_ = 1) or the left (δt_L_ = 1) target. When the working-memory module was added, the sensory evidence stored in it and used as input in the decision-making process was estimated as:

$$\frac{{de}_{leak}}{dt}=\frac{de}{dt}-L_{e}\;e_{leak}$$

where *L*_e_ was the leak term for the working memory. The value of e_leak_ was only updated after the jump of each token and remained with that value until the next token’s jump occurred [36].

#### Decision-making models

We implemented two different models of decision making that have been widely accepted and used to analyze behavior and neural data observed in previous research [7, 41, 44–47]: the EAM and the UGM.

The EAM was implemented with the following dynamics:

$$x_{t+\Delta t}=x_{t}+\nu e_{t}\Delta t+s\xi \sqrt{\Delta t}$$

where *x* describes the state of the process at time t and t+Δt, ν is the drift rate, *e* is the sensory evidence provided (by either the working-memory module or the visual input) to make a decision, Δt is the step size of the simulation, s is a scaling factor and ξ is an independent and identically distributed random sample taken from a standard normal distribution. In each simulated trial, the initial value of *x* was set to $\frac{\theta }{2}$, where θ reflects the difference between decision boundaries, and the drift rate ν was sampled from a normal distribution with standard deviation η and mean ν. In our simulations, the step size Δt was set to 10 ms and s was arbitrarily set to 0.1. In the EAM, a decision was considered to be made when x>θ or x<0 and the corresponding decision time was estimated as $t-\frac{\Delta t}{2}$.

The dynamics of the UGM differed from the EAM in a leaky term and an urgency signal:

$$x_{t+\Delta t}=\frac{\tau }{\tau +\Delta t}x_{t}+\frac{\Delta t}{\tau +\Delta t}(\nu e_{t}\Delta t+s\xi \sqrt{\Delta t})$$

where ν, η and Δt are parameters defined as in the EAM. The leaky term was implemented as a low-pass filter with a time constant given by *τ* (100 ms). The instantaneous value of the accumulation of evidence was multiplied by an urgency signal, which was defined as the time passed since the start of the decision-making process (*u*_*t*_ *= t*). In the UGM, the decision was considered to be made when *x*_*t*+Δ*t*_*u*_*t*+Δ*t*_>*θ* or *x*_*t*+Δ*t*_*u*_*t*+Δ*t*_<−*θ* and the corresponding decision time was estimated as $t-\frac{\Delta t}{2}$.

In both models, the parameters ν, η and θ are free parameters estimated with a fitting procedure that optimized the goodness of fit.

### Estimation of model parameters

The model parameters (ν, η and θ) were estimated independently for each model to fit each participant’s data. To do that, we used quantile maximum products estimation (QMPE) [48]. For that, the decision times of the experimental data were sorted into quantiles, divided into correct and error responses. The QMPE estimates the similarity between experimental and simulated data by comparing the proportion of data that belong to each quantile. The search of parameters to optimize goodness of fit was performed with the differential evolution algorithm [49,50]. We defined wide boundaries for each parameter and used 100 particles for 500 search iterations. This procedure of parameters’ estimation was repeated 5 times to avoid local maxima. Model predictions were evaluated using Monte Carlo simulation with 10,000 replicates per experimental condition. At the search termination point, data was simulated with the set of parameters that had the highest goodness of fit and with a number of trials that matched those in the experimental data.

Data fitting was performed using all (correct and error) easy, ambiguous, bias-for, and bias-against trials.

### Statistical tests

The normality of the datasets was tested using the Shapiro–Wilk test.

Bayes Factors [51] were calculated by using a Cauchy distribution with a scale factor of 3 for DTs and a scale factor of 0.707 for SPs, since big and small differences were expected, respectively. A Bayes Factor greater than 3 or smaller than 1/3 indicated evidence for the alternative or the null hypothesis, respectively [52].

## References

[1] Smith PL, Ratcliff R. Psychology and neurobiology of simple decisions. Trends Neurosci. 2004;27:161–8. doi: 10.1016/j.tins.2004.01.006 15036882

[2] Gold JI, Shadlen MN. The neural basis of decision making. Annu Rev Neurosci. 2007;30:535–74. doi: 10.1146/annurev.neuro.29.051605.113038 17600525

[3] Platt ML, Glimcher PW. Neural correlates of decision variables in parietal cortex. Nature. 1999;400:233–8. doi: 10.1038/22268 10421364

[4] Cisek P, Kalaska J. Neural correlates of reaching decisions in dorsal premotor cortex: specification of multiple direction choices and final selection of action. Neuron. 2005;45:801–14. doi: 10.1016/j.neuron.2005.01.027 15748854

[5] Thura D, Cisek P. Deliberation and commitment in the premotor and primary motor cortex during dynamic decision making. Neuron. 2014;81:1401–16. doi: 10.1016/j.neuron.2014.01.031 24656257

[6] Marcos E, Pani P, Brunamonti E, Deco G, Ferraina S, Verschure P. Neural variability in premotor cortex is modulated by trial history and predicts behavioral performance. Neuron. 2013;78:249–55. doi: 10.1016/j.neuron.2013.02.006 23622062

[7] Stone M. Models for choice-reaction time. Psychometrika. 1960;25:251–60. doi: 10.1007/BF02289729

[8] Van Zandt T, Colonius H, Proctor R. A comparison of two response time models applied to perceptual matching. Psychon Bull Rev. 2000;7:208–56. doi: 10.3758/bf03212980 10909132

[9] Churchland A, Kiani R, Chaudhuri R, Wang X-J, Pouget A, Shadlen M. Variance as a signature of neural computations during decision making. Neuron. 2011;69:818–31. doi: 10.1016/j.neuron.2010.12.037 21338889 PMC3066020

[10] Bollimunta A, Totten D, Ditterich J. Neural dynamics of choice: single-trial analysis of decision-related activity in parietal cortex. J Neurosci. 2012;32:12684–701. doi: 10.1523/JNEUROSCI.5752-11.2012 22972993 PMC6703806

[11] Brown S, Heathcote A. The simplest complete model of choice response time: Linear ballistic accumulation. Cogn Psychol. 2008;57:153–78. doi: 10.1016/j.cogpsych.2007.12.002 18243170

[12] Cassey P, Heathcote A, Brown S. Brain and Behavior in Decision-Making. PLoS Comput Biol. 2014;10:e1003700. doi: 10.1371/journal.pcbi.1003700 24991810 PMC4081035

[13] Dutilh G, Annis J, Brown S, Cassey P, Evans N, Grasman R, et al. The quality of response time data inference: a blinded, collaborative assessment of the validity of cognitive models. Psychon Bull Rev. 2019;26:1051–69. doi: 10.3758/s13423-017-1417-2 29450793 PMC6449220

[14] Churchland A, Kiani R, Shadlen M. Decision-making with multiple alternatives. Nat Neurosci. 2008;11:693–702. doi: 10.1038/nn.2123 18488024 PMC2453226

[15] Cisek P, Puskas G, El-Murr S. Decisions in changing conditions: the urgency-gating model. J Neurosci. 2009;29:11560–71. doi: 10.1523/JNEUROSCI.1844-09.2009 19759303 PMC6665752

[16] Thura D, Beauregard-Racine J, Fradet C-W, Cisek P. Decision making by urgency gating: theory and experimental support. J Neurophysiol. 2012;108:2912–30. doi: 10.1152/jn.01071.2011 22993260

[17] Carland M, Marcos E, Thura D, Cisek P. Evidence against perfect integration of sensory information during perceptual decision making. J Neurophysiol. 2016;115:015–930. doi: 10.1152/jn.00264.2015 26609110

[18] Hawkins G, Wagenmakers E-J, Ratclif R, Brown S. Discriminating evidence accumulation from urgency signals in speeded decision making. J Neurophysiol. 2015;114:40–7. doi: 10.1152/jn.00088.2015 25904706 PMC4495756

[19] Trueblood J, Heathcote A, Evans N, Holmes W. Urgency, leakage, and the relative nature of information processing in decision-making. Psychol Rev. 2021;128:160–86. doi: 10.1037/rev0000255 32852976

[20] Brunton BW, Botvinick MM, Brody CD. Rats and humans can optimally accumulate evidence for decision-making. Science. 2013;340:95–8. doi: 10.1126/science.1233912 23559254

[21] Piet AT, El Hady A, Brody CD. Rats adopt the optimal timescale for evidence integration in a dynamic environment. Nat Commun. 2018;9:4265. doi: 10.1038/s41467-018-06561-y 30323280 PMC6189050

[22] Hanks TD, Kopec CD, Brunton BW, Duan CA, Erlich JC, Brody CD. Distinct relationships of parietal and prefrontal cortices to evidence accumulation. Nature. 2015;520:220–3. doi: 10.1038/nature14066 25600270 PMC4835184

[23] Evans N, Hawkins G, Boehm U, Wagenmakers E-J, Brown S. The computations that support simple decision-making: A comparison between the diffusion and urgency-gating models. Sci Rep. 2017;7:16433. doi: 10.1038/s41598-017-16694-7 29180789 PMC5703954

[24] Winkel J, Keuken M, van Maanen L, Wagenmakers E-J, Forstmann B. Early evidence affects later decisions: Why evidence accumulation is required to explain response time data. Psychon Bull Rev. 2014;21:777–84. doi: 10.3758/s13423-013-0551-8 24395093

[25] Britten K, Shadlen M, Newsome W, Movshon J. The analysis of visual motion: a comparison of neuronal and psychophysical performance. J Neurosci. 1992;12:4745–65. doi: 10.1523/JNEUROSCI.12-12-04745.1992 1464765 PMC6575768

[26] Britten K, Shadlen M, Newsome W, Movshon J. Responses of neurons in macaque MT to stochastic motion signals. Vis Neurosci. 1993;10:1157–69. doi: 10.1017/s0952523800010269 8257671

[27] Shadlen M, Newsome W. Motion perception: seeing and deciding. Proc Natl Acad Sci USA. 1996;93:628–33. doi: 10.1073/pnas.93.2.628 8570606 PMC40102

[28] Shadlen M, Newsome W. Neural basis of a perceptual decision in the parietal cortex (area lip) of the rhesus monkey. J Neurophysiol. 2001;86:1916–36. doi: 10.1152/jn.2001.86.4.1916 11600651

[29] Roitman JD, Shadlen MN. Response of neurons in the lateral intraparietal area during a combined visual discrimination reaction time task. J Neurosci. 2002;22:9475–89. doi: 10.1523/JNEUROSCI.22-21-09475.2002 12417672 PMC6758024

[30] Huk A, Shadlen M. Neural activity in macaque parietal cortex reflects temporal integration of visual motion signals during perceptual decision making. J Neurosci. 2005;25:10420–36. doi: 10.1523/JNEUROSCI.4684-04.2005 16280581 PMC6725829

[31] Zhang S, Lee M, Vandekerckhove J, Maris G, Wagenmakers E-J. Time-varying boundaries for diffusion models of decision making and response time. Front Psychol. 2014;5:1364. doi: 10.3389/fpsyg.2014.01364 25538642 PMC4260487

[32] Milosavljevic M, Malmaud J, Huth A, Koch C, Rangel A. The Drift Diffusion Model can account for the accuracy and reaction time of value-based choices under high and low time pressure. Judgm Decis Mak. 2010;5:437–49. doi: 10.2139/ssrn.1901533

[33] Drugowitsch J, Moreno-Bote R, Churchland A, Shadlen M, Pouget A. The cost of accumulating evidence in perceptual decision making. J Neurosci. 2012;32:3612–28. doi: 10.1523/JNEUROSCI.4010-11.2012 22423085 PMC3329788

[34] Carland M, Thura D, Cisek P. The urgency-gating model can explain the effects of early evidence. Psychon Bull Rev. 2015;22:1830–8. doi: 10.3758/s13423-015-0851-2 26452377

[35] Marcos E, Tsujimoto S, Genovesio A. Event- and time-dependent decline of outcome information in the primate prefrontal cortex. Sci Rep. 2016;6:25622. doi: 10.1038/srep25622 27162060 PMC4861909

[36] Kiani R, Churchland A, Shadlen M. Integration of direction cues is invariant to the temporal gap between them. J Neurosci. 2013;33:16483–9. doi: 10.1523/JNEUROSCI.2094-13.2013 24133253 PMC3797371

[37] Marcos E, Cos I, Girard B, Verschure PFMJ. Motor cost influences perceptual decisions. PLoS ONE. 2015;10:e0144841. doi: 10.1371/journal.pone.0144841 26673222 PMC4684499

[38] Hagura N, Haggard P, Diedrichsen J. Perceptual decisions are biased by the cost to act. eLife. 2017;6:e18422. doi: 10.7554/eLife.18422 28219479 PMC5319835

[39] Marcos E, Genovesio A. Determining monkey free choice long before the choice is made: the principal role of prefrontal neurons involved in both decision and motor processes. Front Neural Circuits. 2016;10:75. doi: 10.3389/fncir.2016.00075 27713692 PMC5031774

[40] Marcos E, Genovesio A. Interference between Space and Time Estimations: From Behavior to Neurons. Front Neurosci. 2017;11:631. doi: 10.3389/fnins.2017.00631 29209159 PMC5702290

[41] Mazurek M, Roitman J, Ditterich J, Shadlen M. A Role for neural integrators in perceptual decision making. Cereb Cortex. 2003;13:1257–69. doi: 10.1093/cercor/bhg097 14576217

[42] Latimer KW, Yates JL, Meister MLR, Huk AC, Pillow JW. Single-trial spike trains in parietal cortex reveal discrete steps during decision-making. Science. 2015;349:184–7. doi: 10.1126/science.aaa4056 26160947 PMC4799998

[43] Thura D, Cisek P. The basal ganglia do not select reach targets but control the urgency of commitment. Neuron. 2017;95:1160–70. doi: 10.1016/j.neuron.2017.07.039 28823728

[44] Laming D. Information Theory of Choice-Reaction Times: London Academic; 1968.

[45] Ratcliff R. A theory of memory retrieval. Psychol Rev. 1978;85:59–108. doi: 10.1037/0033-295X.85.2.59

[46] Usher M, McClelland J. The time course of perceptual choice: the leaky, competing accumulator model. Psychol Rev. 2001;108:550–92. doi: 10.1037/0033-295x.108.3.550 11488378

[47] Bogacz R, Gurney K. The basal ganglia and cortex implement optimal decision making between alternative actions. Neural Comput. 2007;19:442–77. doi: 10.1162/neco.2007.19.2.442 17206871

[48] Heathcote A, Brown S, Mewhort D. Quantile maximum likelihood estimation of response time distributions. Psychon Bull Rev. 2002;9:394–401. doi: 10.3758/bf03196299 12120806

[49] Ardia D, Mullen K, Peterson B, Ulrich J. DEoptim: Differential Evolution in R [Computer software manual]. Retrieved from http://CRAN.Rproject.org/packageDEoptim (R package version 2.2–2). 2013. doi: 10.32614/RJ-2011-005

[50] Mullen K, Ardia D, Gil D, Windover D, Cline J. DEoptim: An R package for global optimization by differential evolution. J Stat Softw. 2011;40:1–26. doi: 10.18637/jss.v040.i06

[51] Rouder JN, Speckman PL, Sun D, Morey RD, Iverson G. Bayesian t tests for accepting and rejecting the null hypothesis. Psychon Bull Rev. 2009;16:225–37. doi: 10.3758/PBR.16.2.225 19293088

[52] Jeffreys H. The theory of probability: Oxford: Oxford University Press; 1961.

